# Quantification of Replacement Fibrosis in Aortic Stenosis: A Narrative Review on the Utility of Cardiovascular Magnetic Resonance Imaging

**DOI:** 10.3390/diagnostics14212435

**Published:** 2024-10-31

**Authors:** Megan R. Rajah, Anton F. Doubell, Philip G. Herbst

**Affiliations:** Division of Cardiology, Department of Medicine, Faculty of Medicine and Health Sciences, Stellenbosch University and Tygerberg Hospital, Cape Town 7505, South Africa

**Keywords:** late gadolinium enhancement, myocardial fibrosis, replacement fibrosis, scar quantification, LGE quantification, aortic stenosis

## Abstract

Aortic stenosis (AS) is associated with the development of replacement myocardial fibrosis/scar. Given the dose-dependent relationship between scar and clinical outcomes after aortic valve replacement (AVR) surgery, scar quantity may serve as an important risk-stratification tool to aid decision-making on the optimal timing of AVR. Scar is non-invasively assessed and quantified by cardiovascular magnetic resonance (CMR) imaging. Several quantification techniques exist, and consensus on the optimal technique is lacking. These techniques range from a visual manual method to fully automated ones. This review describes the different scar quantification techniques used and highlights their strengths and shortfalls within the context of AS. The two most commonly used techniques in AS include the semi-automated signal threshold versus reference mean (STRM) and full-width half-maximum (FWHM) techniques. The accuracy and reproducibility of these techniques may be hindered in AS by the coexistence of diffuse interstitial fibrosis and the presence of relatively small, non-bright scars. The validation of these techniques against histology, which is the current gold standard for scar quantification in AS, is limited. Based on the best current evidence, the STRM method using a threshold of three standard deviations above the mean signal intensity of remote myocardium is recommended. The high reproducibility of the FWHM technique in non-AS cohorts has been shown and merits further evaluation within the context of AS. Future directions include the use of quantitative T1 mapping for the detection and quantification of scar, as well as the development of serum biomarkers that reflect the fibrotic status of the myocardium in AS.

## 1. Introduction

Aortic stenosis (AS) remains one of the most common valve conditions worldwide, and severe disease is associated with significant mortality compared to that of the general population [1,2,3,4]. To date, only one mortality-modifying therapy is available–aortic valve replacement (AVR), which is not without risk [1,2,3,5]. The decision to replace an aortic valve, therefore, requires a careful balance of risk versus benefit and can be challenging in the face of an often technically demanding echocardiography study coupled with having to consider new and constantly evolving evidence.

The American Heart Association (AHA) and European Society of Cardiology (ESC) guidelines agree that AVR is indicated in severe, symptomatic AS and in severe asymptomatic AS where there is evidence of left ventricular (LV) systolic dysfunction [6,7,8]. In those with paradoxical low gradient AS, severe asymptomatic AS without LV systolic dysfunction and moderate AS, the decision to intervene remains uncertain despite evidence of increased mortality associated with these conditions [6,7,9,10,11,12]. Furthermore, there is evidence that even in those groups that do qualify for AVR by current guideline criteria, complete reversal of the adverse structural and functional LV changes is not always achievable [13,14]. Several additional parameters have been developed to further risk stratify patients who fall short of guideline-recommended indications for valvular intervention, including the use of NT-pro BNP, global longitudinal myocardial strain using speckle-tracking echocardiography, very severe elevation in transvalvular gradients and the presence and quantity of myocardial fibrosis [6,15,16,17].

Myocardial fibrosis in AS is well-described. The first reports of myocardial fibrosis in AS were made on histopathology via endomyocardial biopsy, which remains the gold standard technique for the detection and quantification of myocardial fibrosis [18]. Two distinct patterns of fibrosis are described from histopathology: (i) diffuse/reactive interstitial fibrosis, which comprises loose collagen bands surrounding bundles of cardiomyocytes in a diffuse pattern and (ii) focal replacement fibrosis, which is characterized by a dense focal region of collagen deposited in areas of cardiomyocyte loss [19,20]. In AS without concurrent myocardial infarction, the former is thought to precede the latter and may be reversible [19]. Replacement fibrosis, on the other hand, is understood to represent permanent structural damage with strong evidence to suggest that, also, in the context of severe AS, it is associated with worse outcomes despite intervention with AVR [14,21,22,23,24,25,26,27,28,29].

Myocardial fibrosis has been shown to independently predict both functional recovery and mortality after AVR [24,25,26,27,28]. Several studies have also shown a dose-dependent relationship between replacement fibrosis and outcomes in AS [21,24,25,26,28], e.g., Musa et al. demonstrated that for every 1% increase in scar, there was an 8% increase in cardiac mortality and an 11% increase in all-cause mortality [25]. This suggests that not only the presence of scar in AS but also, the amount of myocardial scar may serve as an important parameter in risk stratifying these patients. Although there are diagnostic tools available to incorporate the use of this parameter into clinical practice, consensus is lacking on how best to assess and quantify fibrosis.

Endomyocardial biopsy is considered the gold standard for the detection and quantification of myocardial fibrosis but comes with disadvantages [17,21,28,30,31]. It is invasive in nature, lacks whole-heart representation and is prone to sampling error [17,21,28,31]. Cardiovascular magnetic resonance (CMR) imaging, on the other hand, can detect both patterns of fibrosis, is non-invasive, and offers a more complete visualization of the entire heart, unlike endomyocardial biopsy [15,16,17,31]. A variety of post-processing techniques for the quantification of replacement fibrosis on CMR have been developed, but due to the lack of standardization, this has yet to be incorporated into current practice guidelines [32].

This narrative review aims to describe the CMR techniques used to image replacement fibrosis, the post-processing methods used for its quantification, and the validation and implementation of these methods in the context of AS. It also aims to offer a perspective on the path towards reaching a consensus and future directions of fibrosis quantification in AS.

### 1.1. CMR Image Acquisition for the Detection of Replacement Fibrosis

**Figure 1:**
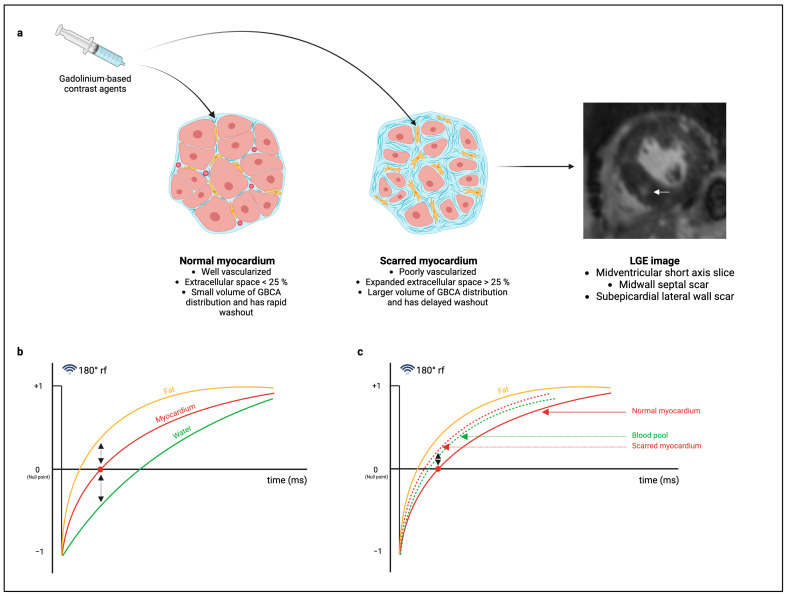
Graphical illustration of scar imaging using late gadolinium enhancement (LGE) imaging. (**a**) The administration of gadolinium-based contrast agents (GBCAs) fills the extracellular space of the normal (left) and scarred (middle) myocardium [19,31,33,34,36]. In scarred myocardium, there is a greater volume of distribution of GBCAs with delayed washout [19,31,33,34,36]. The GBCA in scarred myocardium shortens the T1 relaxation time, the effect of which is encoded into the LGE image, allowing for scar detection, as shown in the LGE image of a patient with severe aortic stenosis (right) [19,31,33,34,36]. The white arrow illustrates a scar in the midwall of the midventricular septum. (**b**) Conventional LGE imaging uses a 180° preparation pulse that inverts the net longitudinal magnetization of fat, myocardium, and blood, each of which passes through a null point upon relaxation [33,34,35,36]. Imaging is performed at the null time for the myocardium, thus creating tissue contrast between bright blood, hypointense myocardium, and bright fat outside the myocardium [33,34,35,36]. (**c**) The presence of GBCAs shortens the T1 relaxation time, resulting in a left shift in the T1 relaxation curves for blood pool and scarred myocardium. Additional tissue contrast is thus achieved between the hypointense normal myocardium (solid red line) and scarred myocardium (dotted red line) [33,34,35,36]. The bright scar has a signal intensity comparable to that of a blood pool (dotted green line) [17,33,34,35,36,39]. Created in Biorender. Rajah, M (2024).

The detection of replacement fibrosis using CMR is achieved using an inversion recovery sequence in the late phase after the administration of gadolinium-based contrast agents (GBCAs) [19,33,34,35,36]. The most widely used contrast agents are class II GBCAs, which are exclusively extracellular, i.e., the agents are incapable of crossing intact cell membranes [17,19,33,34,36,37]. Normal myocardium comprises of tightly packed cardiomyocytes, and up to 75% of the total myocardial volume is therefore intracellular [36,38]. After intravenous gadolinium injection, the agent rapidly fills the relatively small extracellular space and just as rapidly, typically within minutes, is washed out of the myocardium by the capillary network [19,31,33,34,36]. When there is myocardial fibrosis, on the other hand, there is a larger volume of distribution of contrast in the relatively larger extracellular space (expanded interstitium) accompanied by a delayed washout due to the poorly vascularized nature of fibrosis (Figure 1a) [19,31,33,34,36]. Therefore, at the time of acquiring the inversion recovery images, the contrast remains in the expanded extracellular space and its effect on T1 properties is then encoded into the image, allowing for fibrosis detection [19,31,33,34,36].

Conventionally, late gadolinium enhancement (LGE) imaging for replacement fibrosis is performed using a bright-blood T1-weighted inversion recovery sequence. Images are acquired 10–20 minutes after contrast injection [33,34,35,36,39]. A non-slice selective 180° inversion preparation pulse is applied to invert the direction of net longitudinal magnetization (Figure 1b) [33,34,35,36]. The signal intensity of all tissues (fat, myocardium and blood) during relaxation, where the tissue magnetization returns to the baseline, will pass through a null point [33,34,35,36]. Image acquisition, by means of gradient echo or basal steady-state free precession (bSSFP), is then performed at a user-selected time (inversion time) that corresponds specifically to the null point of normal myocardium, i.e., where normal myocardium appears black on the inversion recovery sequence [33,34,35,36]. This strategy is effective at creating tissue contrast between bright blood in the LV cavity, low-signal intensity myocardium, and high-signal intensity fat outside the myocardium [17,33,34,35,36]. Since GBCAs shorten T1 relaxation in direct proportion to the concentration [40], further tissue contrast is achieved between the normal myocardium and scarred myocardium, which appears as high/enhanced signal intensity comparable to that of the blood pool (Figure 1c) [17,33,34,35,36,39].

While this technique is the mostly widely used for scar imaging, challenges with subendocardial scar detection have prompted the development of dark-blood LGE imaging techniques [34,35,36,41,42]. Subendocardial scars, such as those described in myocardial infarction, lead to subendocardial enhancement on conventional LGE imaging. Since scar signal intensity is comparable to that of the blood pool, the interface between a subendocardial scar (when present) and blood pool may be difficult to visualize. Without the ability to recognize this interface, subendocardial scars may be mistaken for blood pool, thus hindering the accurate detection and quantification of these subendocardial scars. Consequently, dark-blood preparation schemes, e.g., FIDDLE and T2 prep-IR/IR-T2 prep, have been developed to address this challenge as described elsewhere [34,35,36,41,42]. Dark-blood LGE imaging may be important in AS as subendocardial scars related to reduced myocardial perfusion reserve have been described [20]. To the best of our knowledge, black blood imaging has yet to be utilized for scar detection in AS.

### 1.2. Post-Processing Methods for the Quantification of Replacement Fibrosis in AS

In the context of AS, a wide range of techniques have been used for the quantification of replacement fibrosis through LGE imaging [32,43]. These range from manual to semi-automated techniques, and more recently, to fully automated ones (Table 1).

#### 1.2.1. Visual LGE Quantification Method

The visual method uses a segmented approach whereby the myocardium is divided into segments (the AHA segmentation model is commonly used), and then by visual assessment, a transmural proportion of replacement fibrosis per segment is estimated by the reader [32,35,63]. The sum of scar per segment then represents the total percentage of scar in the LV, and using the LV mass, a fibrotic mass in grams is derived [32,35,63]. Recent developments in post-processing software have also generated tools to quantify replacement fibrosis based on a manual user-adjusted enhancement overlay. The visual methods are the simplest and have been shown to be reproducible in other pathologies, e.g., myocardial infarction [35,63], but remain the least frequently applied in the context of AS.

#### 1.2.2. Signal Threshold Versus Reference Mean (STRM) Method

The most frequently used method in AS is the semi-automated signal threshold versus reference mean (STRM) technique. This method requires the reader to define endocardial and epicardial borders for every slice on a short-axis stack, covering from the base to the apex of the LV [32]. The reader then contours a region of remote (normal) myocardium on each slice of the short-axis stack [32]. The software uses this region of interest to estimate the mean signal intensity (and its standard deviation) of normal myocardium for each slice [32]. A reader-selected threshold (two to six standard deviations above the mean signal intensity of remote myocardium, depending on the pathology and evidence followed) is then used to identify regions of enhanced signal intensity within the traced endo- and epicardial borders, enabling the software to generate a total percentage replacement fibrosis and a fibrotic mass in grams [32].

**Figure 2:**
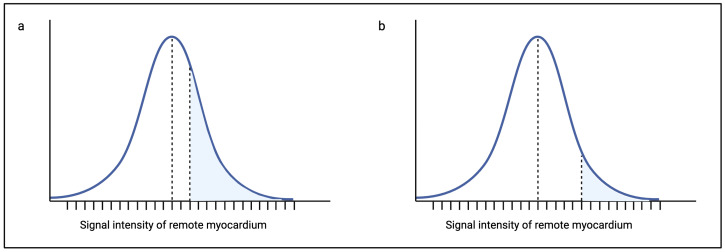
Bell curve graphs showing the range of myocardial signal intensities and application of the STRM technique for replacement fibrosis quantification. (**a**) Signal intensities higher than two standard deviations above the mean signal intensity of remote myocardium are considered fibrosis. The region highlighted in blue represents the range of signal intensities included as fibrosis. (**b**) Signal intensities higher than five standard deviations above the mean signal intensity for remote myocardium are considered fibrosis. The region highlighted in blue represents the range of signal intensities included as fibrosis. At five standard deviations above the mean signal intensity of remote myocardium, the range of signal intensities considered as fibrosis is less than the range at two standard deviations, thus introducing the risk of scar underestimation.

The STRM method is the most frequently used method for scar quantification in AS. The range of standard deviations that are used varies between two and six standard deviations (Table 1). To date, no consensus exists on the optimal number of standard deviations to be used, and this is an important limitation of the method as the amount of scar quantified for a single patient may vary significantly at two standard deviations above the mean for remote myocardium versus five standard deviations [43]. As depicted in Figure 2, the range of signal intensities that would be accepted as scar at two standard deviations above the mean is larger than the range at five standard deviations for the same patient. In practice, this leads to a higher percentage of fibrosis in the two standard deviation group compared to the five standard deviation group. This variation has, in fact, been illustrated in several non-AS cohorts and is discussed later in this review [20,43,64,65,66,67].

The choice of threshold in terms of the optimal number of standard deviations (two to six standard deviations) above remote myocardium is tricky in the context of AS for several reasons. The coexistence of diffuse interstitial fibrosis and replacement fibrosis is well described in AS [17,19,20,39,60,68]. Since interstitial fibrosis resides in the extracellular space, it too enhances during the late gadolinium phase, resulting in challenges with replacement fibrosis quantification using the STRM method. At the very outset, the diffuse distribution of interstitial fibrosis renders the selection of a region of normal remote myocardium a challenge [43]. Furthermore, the STRM method relies on a normal Gaussian distribution of signal intensities for the estimation of a standard deviation, which may be the distribution pattern for normal remote myocardium, but this does not necessarily hold true for myocardium that contains non-uniform, excessive interstitial fibrosis. Additionally, the variable density of interstitial fibrosis from segment to segment ultimately broadens the range of normal myocardial signal intensities even further [20], potentially leading to the overestimation of the absolute amount of replacement fibrosis unless a high standard deviation threshold is chosen.

There are inherent problems with a high standard deviation threshold in AS. Smaller and/or less dense scars are potentially missed. A histological study performed in patients with AS revealed the presence of subendocardial microscars measuring as small as 10 microns or less [20]. The typical voxel sizes in LGE imaging are in the millimeter range, and the signal intensity of a single voxel in the image represents an averaged intensity of all the tissues occupying the voxel [33,38,69]. Therefore, the coexistence of normal myocardium with a microscar in a single voxel is likely to reflect a lower signal intensity than expected, one that is potentially missed by a high standard deviation threshold. Furthermore, even where there are scars large enough to occupy an entire voxel, underestimation at a high standard deviation remains a concern given the relatively bland/non-bright signal intensity of replacement fibrosis/scar in the context of AS compared to the hyperintense signal observed in acute myocardial infarction/myocarditis [38].

#### 1.2.3. Full-Width Half-Maximum (FWHM) Method

The full-width half-maximum (FWHM) technique is also semi-automated but is less frequently used in AS compared to the STRM method. Like the STRM method, endo- and epicardial borders are contoured for every slice in the short-axis LGE stack to cover the whole ventricular myocardium [32]. The technical difference lies in the reader-selected region of interest. Rather than a region of normal myocardium, the region of interest is drawn in an area identified by the operator as replacement fibrosis/scar [32]. The peak signal intensity and half the peak signal intensity of the region of interest/scar are then estimated [32]. All the voxels with a mean signal intensity above half the peak signal intensity of the scar are then considered as replacement fibrosis, and again, the total percentage of fibrosis as well as the mass in grams is estimated. While promising, the technique is not without challenges [32].

As for the STRM technique, the relatively bland signal intensity of replacement fibrosis in AS, together with the presence of background diffuse interstitial fibrosis, brings forth a challenge with this technique. The non-bright signal intensity of replacement fibrosis in AS limits/lowers the peak signal intensity of the scar and, therefore, the half-maximum point. Coupled with the higher mean signal intensity of non-scarred myocardium that results from the presence of diffuse interstitial fibrosis, scar overestimation may be a concern with the FWHM technique in the context of AS [38]. Furthermore, since the region of interest is the scar, small and narrow scars that are sometimes observed in the midwall of the septum may be challenging to accurately contour without sampling adjacent normal myocardium.

Both the STRM and FWHM techniques, while semi-automated, still introduce an element of subjectivity in that the regions of interest are user/reader-selected [32,70]. Nonetheless, while the impact of this subjectivity was demonstrated with the use of the STRM method, its impact using the FWHM technique appears to be less significant. In a hallmark study by Flett et al., systematic bias, when using the STRM method, was driven largely by differences in the region of interest selected by the readers [43]. In this study, bias using the same readers but with the FWHM technique, was significantly lower, and the authors found that the FWHM technique emerged as the only statistically acceptable method for LGE quantification in hypertrophic cardiomyopathy [43,71]. Other studies that have interrogated the use of the FWHM technique in populations of both ischaemic and non-ischaemic pathologies have also highlighted, with consistency, its main strength–reproducibility [64,65,66]. Although this technique improves precision (reproducibility), which may be particularly useful to identify risk change over time, the accuracy of determining the absolute fibrosis burden and, therefore, risk may be sub-optimal for the reasons described.

#### 1.2.4. Fully Automated Method

The new fully automated methods are quickly evolving in the era of machine learning and artificial intelligence but have yet to emerge as a popular technique in the context of AS. The older automated techniques still rely on the reader to define endocardial and epicardial borders. Newer techniques, on the other hand, have incorporated deep machine learning to take over this task with high accuracy [70]. The common benefit of both the old and new automated techniques is that they do not require the user to select a region of remote myocardium or enhanced myocardium for fibrosis quantification, thus removing the largest source of subjectivity associated with the semi-automated techniques [70]. A histogram containing two populations of signal intensities—one for normal myocardium and one for enhanced myocardium—is typically generated using this technique [70]. Through a series of mathematical/statistical analyses, an optimal threshold signal intensity that separates these populations is determined and used to identify and quantify areas of enhancement [70].

While the fully automated method offers the advantage of minimal bias, its disadvantage is that high precision may be achieved at the cost of accuracy. The fully automated technique is not capable of differentiating artifacts from true signal enhancement on LGE images. For example, phase-encoded ghosting artifacts in patients with poor breath-holding during image acquisition may result in the presence of a hyperintense signal within the endo- and epicardial borders of the myocardium (misregistered signal from the chest wall). This signal is likely to be misinterpreted as a scar, leading to scar overestimation.

### 1.3. Validation of LGE Quantification

The popularity of CMR has grown over the years, and LGE imaging is increasingly recognized as the non-invasive gold standard for myocardial fibrosis detection [15,31,39]. As described, several LGE post-processing techniques have been developed for the discernment and quantification of myocardial fibrosis [32]. However, no consensus exists as to which technique yields the optimal result. Establishing a consensus is necessary given the wide variation in quantification results generated from the different methods described. In cohorts of hypertrophic cardiomyopathy and acute/chronic myocardial infarction, large variations have been shown using different techniques [43,64]. Compared to the six standard deviation method, Spiewak et al. demonstrated a nearly eight-fold increase in LGE mass using the one standard deviation method [64]. Similarly, Flett et al. showed a two-fold increase in LGE mass using the two standard deviation method compared to the FWHM technique [43].

Although invasive, histology remains the true gold standard for myocardial fibrosis quantification and is considered the optimal tool for the validation of CMR-based fibrosis quantification methods [72,73]. Studies comparing LGE quantification to histology in AS are, however, rare. Furthermore, the few studies that have tested the correlation between LGE-measured replacement fibrosis and histology appear to have conflicting results. A good correlation between histology and LGE quantification was found in two studies [20,28]. Both studies utilized the STRM method but at different thresholds (three standard deviations and a modified two standard deviation threshold that further incorporated the signal intensity of air). A further two studies that compared LGE-derived fibrosis to histology showed a poor correlation between histology and LGE quantification [40,61]. One of these studies used the STRM method at a threshold of 2.4 standard deviations above remote myocardium, and in the other, the method of LGE quantification was not reported.

There are a few considerations regarding the use of endomyocardial biopsy for the validation of LGE quantification. A gradient of myocardial fibrosis was demonstrated by Treibel et al. in a cohort of AS patients such that the maximum fibrosis was observed at the base, reducing towards the apex of the LV and decreasing from subendocardium to subepicardium [20]. This group also showed that the choice of biopsy instrument influenced the integrity of the biopsy specimen. More specifically, the use of a scalpel often preserved the subendocardial layer, resulting in a better correlation between LGE quantification and subendocardium-containing specimens versus those specimens where the subendocardium was no longer intact/present [20]. The presence/integrity of the subendocardial layer in other histological studies of AS was not always reported, and the impact of this on the accuracy of correlation analyses must be considered.

A variety of histological techniques are available for the quantification of myocardial fibrosis. Commonly used histological stains include the haemotoxylin and eosin (H&E) stain, Masson’s Trichrome stain, and the Picrosirius Red stain–utilization of which varies amongst different institutions [19,74,75]. Although limited, current evidence suggests that the different stains perform equally well in terms of fibrosis quantification [74]. It has, however, been noted that these stains are not necessarily specific to collagen fibers, possibly leading to overestimation [75]. Whether diffuse interstitial fibrosis and replacement fibrosis have been quantified as two separate entities is unclear in most studies, with the exception of work by Treibel et al., where replacement fibrosis was specifically defined on histology and quantified separately to what was identified as diffuse interstitial fibrosis [20]. This is important given the fact that LGE is used specifically for the detection and quantification of replacement fibrosis.

Endomyocardial biopsy specimens are usually sampled from the basal right ventricular septum and, therefore, do not represent the entirety of the LV [72,73]. Given that a fibrotic gradient exists in AS [20], the percentage of fibrosis estimated from a single myocardial segment may not reflect the percentage of fibrosis across the remaining 16 segments. Although diffuse, replacement fibrosis in AS is also patchy [21]. Therefore, endomyocardial biopsy may even miss scar entirely in some cases. The thickness of the sampled endomyocardium has also been shown to play a role in the quantification of fibrosis on histology, therefore requiring strict endomyocardial sampling standardization for valid correlation analyses between histology and LGE-based fibrosis quantification to be performed [74].

Whole-heart analysis may circumvent the issue of sampling representation, but this is a challenging task in the context of AS. One whole-heart study exists in a non-AS cohort where the different LGE quantification methods were analyzed against 11 whole explanted hearts [67]. The outcome of this analysis was that the STRM method using six standard deviations had the closest correlation to whole-heart histology [67]. The study population comprised mostly of dilated cardiomyopathies (DCMs), where some similarities with AS exist in terms of the presence of diffuse interstitial fibrosis and non-bright scars. However, a large proportion of DCM patients have no replacement scarring, and the typical form of replacement scar in DCM (thin midwall scarring) is not typical of AS [76]. In the latter, small areas of focal scar are often observed. As discussed, small focal scars may be missed by high standard deviation thresholds.

### 1.4. Towards a Consensus

Envisaging how the parameter of LGE quantity should be used in clinical practice may be a useful starting point towards reaching a consensus on which LGE quantification method to use. If accuracy is the target, then further validation studies using histology are required, and a standardized validation protocol is recommended. Biopsy specimens with preservation of the subendocardial layer are necessary for a valid fibrosis assessment in AS. As shown by Treibel et al., biopsies taken with a scalpel might improve the success rate of this [20]. The histological protocol should also be optimized in terms of the size and thickness of the specimen to be examined, the choice of staining and quantification technique used, and definitions for replacement versus interstitial fibrosis, which should be reported as two separate entities. Given the limitations of the current histological methods used to quantify fibrosis, more modern histological techniques, such as immunohistochemistry with fluorescently tagged anti-collagen antibodies combined with confocal/super-resolution microscopy are perhaps more useful for the purpose of validation studies [77].

Post-processing of the LGE images also requires precise standardization, e.g., recommendations on whether endo- and epicardial offsets should be used to minimize partial volume effects, the minimum/maximum recommended contour size of the region of interest since this has been shown to influence LGE quantities, which scar should be selected for contouring in slices where more than one scar is observed, and the standard window setting to be used. Most histological validation studies in AS compare a single LGE quantification method. A head-to-head analysis of the different methods against the histology-derived collagen volume fraction is needed and would be most useful. Currently, the best evidence that exists in this regard is that of Treibel et al., who evaluated different thresholds (five, three, and two standard deviations) against histology [20]. As mentioned, the three standard deviation STRM method emerged as the optimal technique [20]. While the performance of the FWHM technique was not reported in this specific study, its high reproducibility from other studies is encouraging and merits testing against the STRM method [64,65,66].

On the other hand, are our efforts better directed towards a method that minimizes bias and ensures reproducibility across centers? Rather than aiming for an accurate, absolute value of replacement fibrosis, it may be clinically as useful to aim for a valid yet arbitrary cutoff based on a pre-agreed methodology that is chosen for precision over accuracy. This could reliably dichotomize patients into high- versus low-risk groups with minimal bias and high reproducibility.

## 2. Future Directions

The development of parametric T1 mapping over recent years has changed the landscape of myocardial tissue characterization for several pathologies using CMR [78]. The pixel-by-pixel measurement of T1 relaxation provides valuable information on the tissue composition of the myocardium, including the presence of myocardial fibrosis [78]. Using pre- and post-contrast T1 mapping, the extracellular volume fraction (ECV) can be estimated and used as a surrogate quantitative marker for diffuse interstitial fibrosis [14,19,20,78]. While ECV is used primarily for the estimation of diffuse interstitial fibrosis [19,20,78], replacement fibrosis is also measurable with this technique. Unlike LGE imaging, T1 mapping is based on a quantitative parameter rather than qualitative signal intensities, affording it more objectivity than LGE quantification. Like endomyocardial biopsy, however, the separation of replacement fibrosis and diffuse interstitial fibrosis may be a challenge and would require a side-by-side analysis using the LGE imaging for further guidance.

In the exciting and rapidly growing field of artificial intelligence, machine learning algorithms for the detection of replacement fibrosis using LGE imaging on CMR are being developed [79]. Few studies have also developed algorithms capable of quantifying scar and have shown promising results with regards to both accuracy and precision [80,81]. It is worthwhile noting that the algorithm training in these studies was based on human analyses that utilized one or more of the quantification techniques described in this article. Establishing a consensus on the optimal technique is, therefore, unavoidable even in the era of artificial intelligence.

A more easily accessible and cheaper alternative to the use of CMR for the evaluation and quantification of myocardial fibrosis in AS may be the use of serum biomarkers that reflect the fibrotic status of the myocardium. Several role players involved in the development, metabolism, and maintenance of myocardial fibrosis have been identified through basic scientific research. These include the superfamily of transforming growth factors (TGF-β), metalloproteinases, and their tissue inhibitors (MMPs and TIMPs, respectively), the C- and N-terminals of procollagens I and III, microRNAs, Galectin-3 and soluble ST2, amongst others [82,83,84]. While the discovery of biomarkers in cell and animal work has been promising, its translation into human studies remains elusive, and further studies are needed.

## 3. Conclusions

The incorporation of LGE quantification into clinical decision-making for AVR in AS may prove valuable, given the dose-dependent relationship between replacement fibrosis and outcomes in this population. While CMR affords us the opportunity to explore this, several quantification methods exist, and consensus on how best to quantify LGE is lacking. This review describes the different post-processing techniques used for the quantification of replacement fibrosis and highlights the strengths and challenges of each technique within the context of AS. The validation of these techniques remains challenging given the described shortfalls of endomyocardial biopsy, which is considered the gold standard for fibrosis quantification. The visual and STRM methods are the most commonly used in AS, and based on the best current evidence, the STRM method using a threshold of three standard deviations above remote myocardium is recommended. Importantly, the impressive reproducibility of the FWHM technique in non-AS cohorts and the objectivity of the fully automated techniques merit further testing in the context of AS.

The quantification of replacement fibrosis using LGE imaging holds promise as a novel risk stratification tool for patients with AS. Standardization of the LGE quantification technique serves as an important step towards incorporating this tool into clinical practice guidelines where its use has the potential to improve patient selection and the optimal timing of AVR, thus improving outcomes.

## Figures and Tables

**Table 1:** Summary of LGE quantification techniques used for replacement fibrosis quantification in aortic stenosis.

Study	LGE Quantification Method	Endomyocardial Biopsy for Validation of LGE Quantification
Weidemann et al. (2009) [21]	Counted segments	Yes
Dusenberry et al. (2014) [44]	Counted segments	No
Chin et al. (2014) [45]	2 SD STRM method	No
Shah et al. (2014) [46]	2 SD STRM method	No
Rudolph et al. (2009) [47]	2 SD STRM method	No
Debl et al. (2006) [48]	2 SD STRM method	No
Barone-Rochette et al. (2014) [23]	2.4 SD STRM method	No
Rajesh et al. (2017) [26]	2.4 SD STRM method and counted segments	No
De Meester de Ravenstein et al. (2015) [40]	2.4 SD STRM method	Yes
Treibel et al. (2018) [20]	3 SD STRM method	Yes
Treibel et al. (2018) [14]	3 SD STRM method	No (Published in previous article)
Everett et al. (2018) [49]	3 SD STRM method	No
Tastet et al. (2020) [50]	3 SD STRM method and FWHM	No
Puls et al. (2020) [13]	3 SD STRM method	No
Child et al. (2018) [51]	4 SD STRM method	No
Singh et al. (2021) [52]	5 SD STRM method	No
Lee et al. (2018) [53]	5 SD STRM method	No
Maltes et al. (2022) [54]	5 SD STRM method	No
Maltes et al. (2023) [55]	5 SD STRM method	No
Hwang et al. (2020) [56]	5 SD STRM method	No
Balčiūnaitė et al. (2021) [57]	5 SD STRM method	No
Hoffmann et al. (2014) [58]	6 SD STRM method	No
Quarto et al. (2012) [27]	FWHM	No
Dweck et al. (2011) [24]	FWHM	No
Musa et al. (2018) [25]	FWHM	No
Fairbairn et al. (2013) [59]	FWHM	No
Everett et al. (2020) [60]	FWHM	No
Azevedo et al. (2010) [28]	Modified 2 SD STRM method	Yes
Maltes et al. (2022) [61]	Unknown	Yes
Kwak et al. (2021) [62]	Unknown	No

LGE: late gadolinium enhancement; SD: standard deviation; STRM: signal threshold versus reference mean; FWHM: full-width half-maximum.

## Data Availability

No new data were created or analyzed in this article. Data sharing is not applicable to this article.

## Abbreviations

AS	aortic stenosis
AVR	aortic valve replacement
AHA	American Heart Association
ESC	European Society of Cardiology
LV	left ventricle/left ventricular
CMR	cardiovascular magnetic resonance imaging
GBCAs	gadolinium-based contrast agents
LGE	late gadolinium enhancement
STRM	signal threshold versus reference mean
SD	standard deviation(s)
FWHM	full-width half-maximum
H&E	haemotoxylin and eosin
DCM	dilated cardiomyopathy
ECV	extracellular volume
